# Comments on “The Impacts of Earthquakes on Air Pollution and Strategies for Mitigation: A Case Study of Turkey”

**DOI:** 10.1007/s11356-024-34152-6

**Published:** 2024-06-29

**Authors:** Kyoo-Man Ha

**Affiliations:** Faculty of Resilience, Rabdan Academy, Abu Dhabi, UAE

Using the case of the Richter scale 7.8 magnitude earthquake in Turkey (as well as Syria) in 2023, Zanoletti and Bontempi (the corresponding author) (2024) investigated earthquake impacts on air pollution levels. Strong earthquakes, according to the researchers, put public health and the ecosystem at risk by releasing hazardous materials into the air, which can include soil and water, such as lead, asbestos, and various toxins. After looking over relevant literature and earlier research, scientists noted that, contrary to popular belief, the impacts of related earthquakes posed a far greater threat to society (Zanoletti and Bontempi 2024).

The majority of earthquake-related research has discussed the subject of building collapse, building codes, anti-seismic structures, building resilience, or else (Mungase et al. 2024). On the contrary, Zanoletti and Bontempi’s research on hazardous material release offered a unique opportunity to understand an unpopular aspect of the earthquake frame. A large number of earthquake researchers have worked hard to address various aspects of building safety. Namely, the topic of hazmat material release around earthquakes will undoubtedly contribute to forming unexpected anti-seismic strategies.

Negative impacts of volcanic ash on local communities were the main topic of discussion among researchers whenever volcanoes erupted nowadays. However, very few scientists referred to the possibility that volcanic ash contributed to the Earth’s temperature falling during climate change (Hagen and Azevedo 2023). In a similar vein, numerous researchers have investigated earthquake impacts on building safety. However, Zanoletti and Bontempi wrote superbly about the detrimental impacts of hazardous material release. In summary, the above two natural hazards were similar in nature, but they had different impacts, such as positive versus negative.

Based on research by Zanoletti and Bontempi, the following four disaster management principles ought to have received more equal support than they do now. First, the hazardous material release should be regarded as the secondary disaster resulting from the occurrence of earthquakes, given that cascading disasters include those extreme events (as the trigger) that unexpectedly generate other disasters in the field of disaster management (Alexander and Pescaroli 2019). People are still affected physically and socially by both earthquakes and the release of hazardous materials (which are cascading disasters).

There are two types of disasters—sudden-onset and slow-onset—depending on how quickly they start (Nguyen et al. 2024). Local communities are typically not given much time to respond to sudden-onset disasters, because they happen so suddenly. On the other hand, slow-onset disasters typically receive little (or less immediate) attention, because they have developed gradually over a region. Both earthquakes (as a sudden-onset disaster) and public health crises from hazardous material release (as a slow-onset disaster) have required tailored strategies during the disaster management cycle.

Regarding the notion that natural disasters are no longer exclusively natural in the modern field of disaster management, the occurrence of earthquakes is not entirely natural either (Deruelle 2023). It indicates that there have been frequent interactions between human factors—political, economic, social, cultural, and other environmental factors—and the occurrence of earthquakes. To be more specific, earthquake impacts, such as chemical material releases and public health emergencies, have been made worse by human activities (e.g., vulnerable assets) or social vulnerabilities (e.g., inequality).

Applications of comprehensive emergency management (CEM) may be necessary when earthquakes occur. The process of CEM encompasses all stakeholders, all hazards, all risks, and phases of the emergency management cycle (i.e., emergency prevention/mitigation, preparedness, response, and recovery) (Jensen and Kirkpatrick 2022). Due to grandiose (or ideal) management, it has been extremely difficult for the field to significantly accomplish the CEM objective. However, the field will proceed in the right direction of disaster management, as long as it theoretically depends on CEM.

## Data Availability

Not applicable.
